# Post-intubation tracheal stenosis in the ICU: diagnosis and treatment

**DOI:** 10.1186/cc9586

**Published:** 2011-03-11

**Authors:** N Makhoul, E Altman, S Croitoru, S Ivry, A Gurevich, S Krimerman, M Croitoru

**Affiliations:** 1 Western Galilee Hospital, Naharya, Israel; 2 Western Galilee Hospital Naharia, Israel; 3 Bnai Zion Medical Center, Haifa, Israel

## Introduction

Prolonged mechanical ventilation of critically ill patients may be complicated by formation of post-intubation tracheal stenosis (PITS) with respiratory disorders of different grades. Critical post-intubation tracheal stenosis (CPITS) may create life-threatening conditions. However, organized teamwork on the ground in the ICU may give positive results.

## Methods

We reviewed retrospectively the medical records of 17 patients admitted to our ICU with PITS and CPITS during 2003 to 2010. Ten of them were males with mean age 68 years old and seven females with mean age 72. In relatively stable patients, computed tomography (CT) and virtual tracheoscopy (VT) were used, followed by rigid (RB) or fiberoptic (FOB) bronchoscopy. In emergency cases we used RB for diagnosis and treatment. All procedures in the operating room were done under general anesthesia, the majority with high-frequency jet ventilation (HFJV).

## Results

In 13 patients PITS had diameter of about 5 to 6 mm and produced dyspnea. Four of 13 patients had soft PITS that were dilated with boogie; in another five patients with hard stenosis, balloon dilation was used. In the remaining four patients with severe respiratory distress, CPITS was diagnosed as having diameter of 3 to 4 mm. Emergency tracheostomy was performed in two patients; excision of large granulations in one case, and intubation with small endotracheal tube after partial dilation in one case.

## Conclusions

Management of PITS in the ICU was beneficial for some of our patients and especially those with CPITS. VT allowed precise measurements of PITS. HFJV created stable conditions for work.

